# New perspectives on an ancient pathogen: thoughts for World Tuberculosis Day 2022

**DOI:** 10.1099/mic.0.001178

**Published:** 2022-03-31

**Authors:** Dany J. V. Beste

**Affiliations:** ^1^​ Department of Microbial Sciences, Faculty of Health and Medical Sciences, University of Surrey, Guildford GU2 7XH, UK

**Keywords:** mycobacteria, tuberculosis, antibiotic resistance, biofilms, metabolism

The last special edition dedicated to mycobacteria was in 2003 and focused on the promise of the post-genomic era to deliver significant advances in mycobacterial research [1]. In the intervening decades we have advanced our understanding about the basic biology of this important group of bacteria. Next-generation sequencing has revolutionized our knowledge of the evolutionary dynamics of mycobacteria, including the development of drug resistance [2]. There have also been innovations in terms of tuberculosis (TB) diagnostics and control measures, including new antibiotics, which have translated into a reduction in cases and death rates from this disease. However, disruption of TB control measures has sadly been amongst the collateral damage of the coronavirus disease 2019 (COVID-19) pandemic, and in 2020 we saw the first increase in TB deaths recorded for over a decade [3]. This is predicted to setback the World Health Organization’s (WHO’s) strategy for reducing the global burden of TB by ~12 years [4]. Mycobacterial research remains massively underfunded, non-TB mycobacterial infections are on the rise and *Mycobacterium bovis* remains a dominant cause of bovine and zoonotic TB worldwide. Despite these impediments, mycobacterial researchers continue to deliver exciting and cutting-edge research, as exemplified in this special issue of *Microbiology*.

Mycobacterial researchers have led the way in the emerging frontiers of pathometabolism and immunometabolism. We now have a much better understanding of the dietary requirements of this pathogen when it is growing within its human host [2] and how *M. tuberculosis* affects its host’s metabolism and immunity. Attention is now being redirected to understanding the battle for metal ions during host pathogen interactions. Understanding metal acquisition by mycobacteria and how metals are used by the host as bacteriostatic/bactericidal weapons is the focus of a review by Serafini and a study by Tamuhla *et al.* [5, 6]. Serafini reviews our limited knowledge of the interplay between central carbon metabolism and metal homeostasis and how this could lead to the development of anti-TB drugs [5]. Tamuhla and colleagues defined new genetic mechanisms by which *M. smegmatis* adapts to low iron stress [6].

Increasing antibiotic resistance (AMR) is a significant impediment to the control of TB and non-TB mycobacterial infections. The WHO deemed *M. tuberculosis* so antibiotic resistant that it was not included in its list of priority antibiotic-resistant pathogens (it is a footnote at the bottom of the widely quoted league table), which has proved unfortunate for the profile of this pathogen. *M. tuberculosis* tragically causes up to 25 % of AMR-associated deaths [7] and therefore surpasses the other pathogens on this list. The UK has pioneered next-generation sequencing for TB diagnostics and drug susceptibility testing. However, such data must be correlated with robust, cost-effective phenotypic methods for assessing drug susceptibility, as developed by CRyPTIC (Comprehensive Resistance Prediction for Tuberculosis: an International Consortium) [8]. Non-TB mycobacteria, including *Mycobacterium abscessus *, are also important human pathogens, which are associated with severe morbidity and mortality and have limited treatment options because of their inherent antibiotic resistance. Consequently, phage therapy is being explored as an alternative to antibiotics to treat TB and non-TB mycobacterial diseases. Joshi *et al.*’s study [9] provides a mechanistic insight into the lysis of mycobacterial cells by studying a mycobacteriophage-encoded endolysin enzyme, whilst S. Singh *et al*. [10] are using a phage genomics approach to identify phage proteins capable of killing mycobacteria. Another exciting strategy is to design adjunctive therapeutics that target error prone DNA repair mechanisms with the goal of preventing the development of AMR. To inform this approach, Amandeep Singh [11] reviews our current understanding of DNA repair systems of mycobacteria.

Treatment of tuberculosis requires a cocktail of drugs to target all mycobacterial populations, including those that are refractory to antibiotic killing because of the specific physiological/phenotypic state of the bacteria. This antibiotic tolerance can affect the whole bacterial population, or a small sub-population known as persisters. Antibiotic-tolerant bacteria are more likely to go on to become genetically resistant and therefore directly contribute to AMR, as reviewed by Mandal *et al*. [12]. This emphasizes the importance of including drug screening methods that test the susceptibility of antibiotic-tolerant populations in our drug discovery programmes [12]. Multiple mechanisms have been proposed to trigger antibiotic tolerance, including, rather ironically, exposure to antibiotics themselves [13]. Biofilm formation is a key survival strategy used by non-TB mycobacteria that is also associated with increased antibiotic tolerance. Borlee *et al.* [14] use compounds secreted by amoeba as a novel approach to disrupt mycobacterial biofilms. Hibernation of ribosomes induced by zinc starvation also results in antibiotic tolerance [15]. Therapies that target the host are another attractive approach to killing antibiotic tolerant populations, as discussed by Rankine-Wilson *et al.* [16].

We have so much more to learn about what is undoubtedly one of the most complex bacterial cell walls, and is also an important drug target because it is intrinsically linked to mycobacterial survival and virulence [2]. The role of the cell wall component phthiocerol dimycocerosates (PDIMs) in mycobacterial pathogenesis is reviewed in this special issue, and this remains highly pertinent, as these lipids are frequently lost during *in vitro* passage [17]. Di Capua *et al.* [18] explore the cell wall epoxy-mycolates. Our understanding of the proteins contained with the mycobacterial membrane has been advanced significantly by cryogenic electron microscopy methods, which have allowed researchers to solve the structure of the type VII protein transport secretion systems, which are central to virulence and crucial for nutrient and metabolite transport across the mycobacterial cell envelope. Lagune *et al.* review where we are in terms of understanding their function in non-TB mycobacteria [19].

Regulation of events within mycobacterial cells is the focus of several papers [20–22]. Insights into how the mycobacterial stress response can be mediated by the two-component transcriptional regulator MtrA and a TetR regulator are pursued [21, 22]. The role of the mycobacterial sepIVA (coil-coiled proteins associated with septation) in the regulation of cell shape and cell wall synthesis is explored by Pickford *et al*. [20]. This work also highlights challenges in reproducing mycobacterial phenotypes between different research groups [20].

There are several papers focused on non-tuberculosis mycobacteria [23–26]. Davarpanah *et al.* [24] demonstrate reservoirs of a variety of opportunistic environmental mycobacteria in dust and soil in Iranian hospitals as potential sources of infection. Another environmental source of non-TB mycobacteria is water, and the mycobacterial thermophile *Mycobacterium hassiacum,* although it rarely infects humans, could be used as an indicator of disinfection success with utility in the hospital environment, and also as a source of thermostable and tractable enzymes for drug design [23]. Mycobacteria are used in bioremediation and Ogawa *et al*. [25] investigate the collaborative degradation of phenanthrene by *Mycobacteria* and *Burkholderia *.

Because of the challenges posed by mycobacterial research (slow growth rate, requirement for high containment, clumping, impenetrable cell wall, etc.), the knowledge and tools available for this group of bacteria previously trailed behind those for other more easily tractable pathogens. However, the last two decades has seen mycobacterial researchers pioneering exciting methodologies, tools and emerging paradigms in host pathogen interactions. The challenge is to translate this research into impactful solutions for the control and prevention of mycobacterial diseases.
